# Bis[*N*-benzyl-2-(quinolin-8-yl­oxy)acetamide]dichloridocopper(II) acetonitrile solvate monohydrate

**DOI:** 10.1107/S1600536810005453

**Published:** 2010-02-13

**Authors:** Yuan Wang, Wei-Na Wu, Rui-Qi Zhao, Ai-Yun Zhang, Bao-Feng Qin

**Affiliations:** a Department of Physics and Chemistry, Henan Polytechnic University, Jiaozuo 454000, People’s Republic of China; b Lanzhou Institute of Chemical Physics, Chinese Academy of Sciences, Lanzhou 730000, People’s Republic of China

## Abstract

In the title complex, [CuCl_2_(C_18_H_16_N_2_O_2_)_2_]·CH_3_CN·H_2_O, the six-coordinated Cu atom is in a distorted octa­hedral geometry with the donor centers of two O atoms and two N atom from two bidentate ligands, and two chloride ions. In the crystal, pairs of inter­molecular N—H⋯ Cl hydrogen bonds form centrosymmetric dimers and inter­molecular O—H⋯ O hydrogen bonds between the ligand and the uncoordinated water mol­ecules link the dimers into chains parallel to the *c* axis.

## Related literature

For the synthesis of *N*-phenyl-2-(quinolin-8-yl­oxy)acetamide, see Wu, Yuan *et al.* (2006 ▶); Wu *et al.* (2008 ▶). For related structures, see: Al-Mandhary & Steel (2002 ▶); Wu, Wang *et al.* (2006 ▶); Zhu *et al.* (2005 ▶).
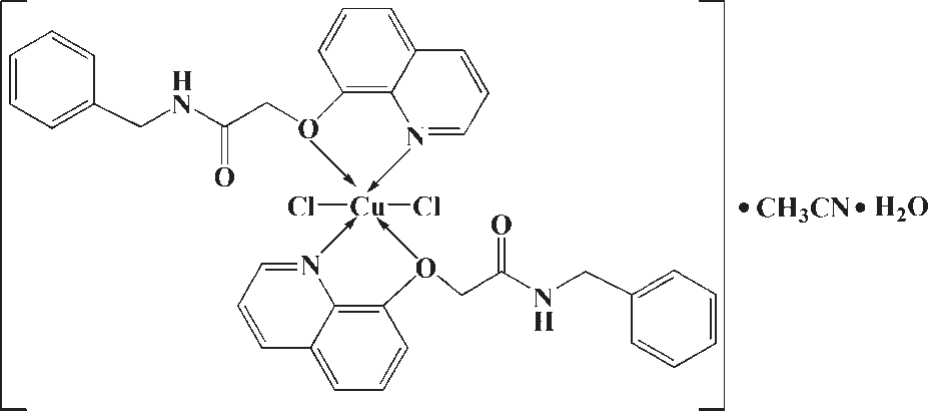


## Experimental

### 

#### Crystal data

[CuCl_2_(C_18_H_16_N_2_O_2_)_2_]·C_2_H_3_N·H_2_O
*M* *_r_* = 778.17
Triclinic, 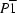

*a* = 10.202 (3) Å
*b* = 13.253 (4) Å
*c* = 14.009 (4) Å
α = 78.927 (3)°
β = 78.995 (3)°
γ = 86.366 (3)°
*V* = 1824.0 (9) Å^3^
*Z* = 2
Mo *K*α radiation
μ = 0.80 mm^−1^
*T* = 293 K
0.31 × 0.21 × 0.13 mm

#### Data collection

Bruker SMART CCD diffractometer
Absorption correction: multi-scan (*SADABS*; Sheldrick, 1996 ▶) *T*_min_ = 0.818, *T*_max_ = 0.902
19499 measured reflections
7457 independent reflections
4374 reflections with *I* > 2σ(*I*)
*R*_int_ = 0.059

#### Refinement

*R*[*F* ^2^ > 2σ(*F* ^2^)] = 0.061
*wR*(*F* ^2^) = 0.186
*S* = 1.03
7457 reflections
464 parameters
9 restraints
H atoms treated by a mixture of independent and constrained refinement
Δρ_max_ = 0.71 e Å^−3^
Δρ_min_ = −0.85 e Å^−3^

### 

Data collection: *SMART* (Bruker, 1997 ▶); cell refinement: *SAINT* (Bruker, 1997 ▶); data reduction: *SAINT*; program(s) used to solve structure: *SHELXS97* (Sheldrick, 2008 ▶); program(s) used to refine structure: *SHELXL97* (Sheldrick, 2008 ▶); molecular graphics: *SHELXTL* (Sheldrick, 2008 ▶); software used to prepare material for publication: *SHELXTL*.

## Supplementary Material

Crystal structure: contains datablocks I, New_Global_Publ_Block. DOI: 10.1107/S1600536810005453/vm2018sup1.cif

Structure factors: contains datablocks I. DOI: 10.1107/S1600536810005453/vm2018Isup2.hkl

Additional supplementary materials:  crystallographic information; 3D view; checkCIF report

## Figures and Tables

**Table 1:** Hydrogen-bond geometry (Å, °)

*D*—H⋯*A*	*D*—H	H⋯*A*	*D*⋯*A*	*D*—H⋯*A*
N2—H2*A*⋯Cl1^i^	0.86	2.35	3.160 (4)	158
N4—H4*A*⋯Cl2	0.86	2.39	3.230 (5)	165
O1*W*—H1*WA*⋯O2	0.85	1.95	2.776 (8)	165
O1*W*—H1*WB*⋯O4^ii^	0.86 (2)	2.2 (2)	2.781 (11)	121 (23)

Symmetry codes: (i) 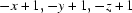; (ii) 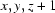.

**Fig. 1.**
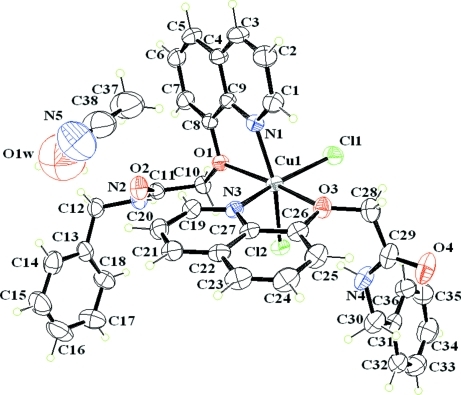
The molecular structure shown with 30% probability displacement ellipsoids.

**Fig. 2.**
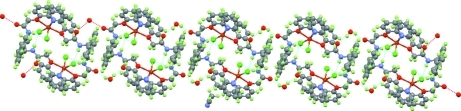
The crystal packing for the title complex via hydrogen bonds (dashed lines).

## supplementary crystallographic information

### Comment

The luminescent properties of the lanthanide complexes with amide type ligands have been investigated in our previous work (Wu, Yuan *et al.*, 2006; Wu *et al.*, 2008). As part of our ongoing studies of the amide type ligands, the title complex was synthesized and characterized by X-ray diffraction.

As shown in Fig. 1, in the title complex, the six-coordinated Cu atom is in a distored octahedral geometry with the donor centers of two O atoms and two N atom from two ligands, and two chloride ions. It is worth noting that the O3 atom of the ligand also participates coordination since the distance of Cu1—O3 are 2.446 (3) Å. A longer Cu—O distance of 2.586 Å has been reported in complex [Cu_3_(L_1_)_2_Cl_6_].2DMF, where L_1_= 1,3,5-tris(2-pyridylmethoxyl)benzene (Wu, G. *et al.* 2006). Other features of the structure are similar to those found in other sixcoordinate copper(II) complexes with similar donor sets (Al-Mandhary *et al.*, 2002; Wu, Wang *et al.*, 2006 & Zhu *et al.*, 2005).

In the crystal, two intermolecular N—H··· Cl hydrogen bonds between two molecules create centrosymmetric dimers. and the dimers are linked into chains via the intermolecular O—H··· O hydrogen bonds between the ligand and the uncoordinated water molecules. Intermolecular N—H··· Cl hydrogen bonds are also present (Fig. 2).

### Experimental

*N*-benzyl-2-(quinolin-8-yloxy)acetamide (Wu, Yuan *et al.*, 2006) (0.292 g, 1 mmol) was dissolved in acetonitrile (10 ml), then an acetonitrile solution (10 ml) containing copper(II) chloride dihydrate (0.179 g, 1 mmol) was added dropwise at room temperature. After stirring for 2 h, the mixture was filtered and set aside to crystallize at room temperature for 10 d, giving blue block crystals.

### Refinement

The C31 phenyl ring was refined as rigid hexagon of 1.39 Å sides as there was a slight spread of C–C distances. The water H-atoms were located in a difference Fourier map and refined with an O—H distance restrain of 0.85 (2) Å. Other H atoms attached to C and N atoms were placed in calculated positions and treated using a riding-model approximation (C—H=0.93 for aromatic ring H atoms with *U*_iso_(H)=1.2Ueq(C); N—H=0.86 with *U*_iso_(H)=1.5Ueq(N)).

### Crystal data

**Table:**

[CuCl_2_(C_18_H_16_N_2_O_2_)_2_]·C_2_H_3_N·H_2_O	*V* = 1824.0 (9) Å^3^
*M**_r_* = 778.17	*Z* = 2
Triclinic, *P*1	*F*(000) = 806
Hall symbol: -P 1	*D*_x_ = 1.417 Mg m^−^^3^
*a* = 10.202 (3) Å	Mo *K*α radiation, λ = 0.71073 Å
*b* = 13.253 (4) Å	θ = 2.0–26.5°
*c* = 14.009 (4) Å	µ = 0.80 mm^−^^1^
α = 78.927 (3)°	*T* = 293 K
β = 78.995 (3)°	Block, blue
γ = 86.366 (3)°	0.31 × 0.21 × 0.13 mm

### Data collection

**Table:**

Bruker SMART CCD diffractometer	7457 independent reflections
Radiation source: fine-focus sealed tube	4374 reflections with *I* > 2σ(*I*)
graphite	*R*_int_ = 0.059
phi and ω scans	θ_max_ = 26.5°, θ_min_ = 2.0°
Absorption correction: multi-scan (*SADABS*; Sheldrick, 1996)	*h* = −12→12
*T*_min_ = 0.818, *T*_max_ = 0.902	*k* = −16→16
19499 measured reflections	*l* = −17→17

### Refinement

**Table:**

Refinement on *F*^2^	Primary atom site location: structure-invariant direct methods
Least-squares matrix: full	Secondary atom site location: difference Fourier map
*R*[*F*^2^ > 2σ(*F*^2^)] = 0.061	Hydrogen site location: inferred from neighbouring sites
*wR*(*F*^2^) = 0.186	H atoms treated by a mixture of independent and constrained refinement
*S* = 1.03	*w* = 1/[σ^2^(*F*_o_^2^) + (0.1001*P*)^2^] where *P* = (*F*_o_^2^ + 2*F*_c_^2^)/3
7457 reflections	(Δ/σ)_max_ = 0.030
464 parameters	Δρ_max_ = 0.71 e Å^−^^3^
9 restraints	Δρ_min_ = −0.85 e Å^−^^3^

### Special details

**Table:**

Geometry. All esds (except the esd in the dihedral angle between two l.s. planes) are estimated using the full covariance matrix. The cell esds are taken into account individually in the estimation of esds in distances, angles and torsion angles; correlations between esds in cell parameters are only used when they are defined by crystal symmetry. An approximate (isotropic) treatment of cell esds is used for estimating esds involving l.s. planes.
Refinement. Refinement of *F*^2^ against ALL reflections. The weighted *R*-factor wR and goodness of fit *S* are based on *F*^2^, conventional *R*-factors *R* are based on *F*, with *F* set to zero for negative *F*^2^. The threshold expression of *F*^2^ > σ(*F*^2^) is used only for calculating *R*-factors(gt) etc. and is not relevant to the choice of reflections for refinement. *R*-factors based on *F*^2^ are statistically about twice as large as those based on *F*, and *R*- factors based on ALL data will be even larger.

### Fractional atomic coordinates and isotropic or equivalent isotropic displacement parameters (Å^2^)

**Table:**

	*x*	*y*	*z*	*U*_iso_*/*U*_eq_	
Cu1	0.70023 (5)	0.29872 (4)	0.40084 (4)	0.04355 (19)	
O1	0.6634 (3)	0.3689 (2)	0.5515 (2)	0.0474 (7)	
O2	0.5770 (4)	0.2507 (2)	0.7300 (2)	0.0658 (9)	
O3	0.7440 (3)	0.1992 (2)	0.2675 (2)	0.0558 (8)	
O4	0.6412 (5)	0.2161 (4)	0.0407 (3)	0.1052 (15)	
N1	0.8765 (3)	0.3017 (3)	0.4417 (3)	0.0461 (8)	
N2	0.4376 (4)	0.3748 (3)	0.7784 (3)	0.0571 (10)	
H2A	0.4049	0.4354	0.7603	0.068*	
N3	0.6864 (3)	0.1485 (3)	0.4624 (3)	0.0450 (8)	
N4	0.5215 (5)	0.2459 (4)	0.1848 (3)	0.0731 (13)	
H4A	0.5265	0.2605	0.2412	0.088*	
C1	0.9814 (5)	0.2679 (4)	0.3872 (4)	0.0631 (13)	
H1	0.9699	0.2367	0.3354	0.076*	
C2	1.1110 (5)	0.2766 (5)	0.4033 (5)	0.0812 (17)	
H2	1.1840	0.2509	0.3635	0.097*	
C3	1.1281 (5)	0.3220 (5)	0.4762 (5)	0.0744 (16)	
H3	1.2141	0.3292	0.4868	0.089*	
C4	1.0206 (5)	0.3584 (4)	0.5365 (4)	0.0588 (13)	
C5	1.0327 (6)	0.4074 (5)	0.6140 (4)	0.0757 (16)	
H5	1.1167	0.4153	0.6277	0.091*	
C6	0.9250 (6)	0.4426 (4)	0.6681 (4)	0.0766 (17)	
H6	0.9347	0.4752	0.7194	0.092*	
C7	0.7977 (5)	0.4320 (4)	0.6499 (4)	0.0639 (14)	
H7	0.7236	0.4577	0.6886	0.077*	
C8	0.7817 (4)	0.3850 (3)	0.5768 (3)	0.0486 (11)	
C9	0.8941 (4)	0.3470 (3)	0.5172 (3)	0.0459 (10)	
C10	0.5454 (4)	0.3984 (3)	0.6105 (3)	0.0491 (11)	
H10A	0.4703	0.3927	0.5788	0.059*	
H10B	0.5504	0.4698	0.6161	0.059*	
C11	0.5224 (4)	0.3329 (3)	0.7126 (3)	0.0471 (10)	
C12	0.3973 (5)	0.3233 (4)	0.8795 (3)	0.0656 (14)	
H12A	0.3773	0.3747	0.9215	0.079*	
H12B	0.4717	0.2808	0.8998	0.079*	
C13	0.2795 (6)	0.2578 (4)	0.8961 (3)	0.0618 (13)	
C14	0.2840 (7)	0.1598 (5)	0.9468 (4)	0.0849 (18)	
H14	0.3629	0.1326	0.9666	0.102*	
C15	0.1726 (10)	0.1004 (6)	0.9691 (6)	0.113 (3)	
H15	0.1765	0.0334	1.0041	0.136*	
C16	0.0591 (9)	0.1384 (6)	0.9409 (6)	0.120 (3)	
H16	−0.0164	0.0984	0.9575	0.143*	
C17	0.0531 (7)	0.2365 (5)	0.8872 (6)	0.102 (2)	
H17	−0.0255	0.2627	0.8664	0.123*	
C18	0.1644 (6)	0.2949 (4)	0.8650 (5)	0.0769 (16)	
H18	0.1613	0.3610	0.8280	0.092*	
C19	0.6631 (4)	0.1215 (3)	0.5579 (4)	0.0518 (11)	
H19	0.6587	0.1728	0.5954	0.062*	
C20	0.6444 (5)	0.0211 (4)	0.6076 (4)	0.0583 (12)	
H20	0.6281	0.0062	0.6764	0.070*	
C21	0.6501 (5)	−0.0535 (4)	0.5556 (4)	0.0599 (13)	
H21	0.6381	−0.1213	0.5880	0.072*	
C22	0.6738 (4)	−0.0304 (3)	0.4537 (4)	0.0534 (12)	
C23	0.6761 (5)	−0.1045 (4)	0.3929 (5)	0.0688 (15)	
H23	0.6622	−0.1732	0.4217	0.083*	
C24	0.6977 (6)	−0.0771 (4)	0.2949 (5)	0.0734 (15)	
H24	0.6991	−0.1270	0.2562	0.088*	
C25	0.7184 (5)	0.0248 (4)	0.2495 (4)	0.0693 (14)	
H25	0.7316	0.0428	0.1809	0.083*	
C26	0.7196 (4)	0.0979 (3)	0.3047 (4)	0.0519 (11)	
C27	0.6934 (4)	0.0720 (3)	0.4085 (3)	0.0446 (10)	
C28	0.7583 (5)	0.2327 (4)	0.1643 (4)	0.0724 (15)	
H28A	0.8260	0.1897	0.1317	0.087*	
H28B	0.7899	0.3025	0.1478	0.087*	
C29	0.6328 (7)	0.2302 (4)	0.1244 (4)	0.0740 (15)	
C30	0.3922 (6)	0.2396 (4)	0.1612 (4)	0.0781 (16)	
H30A	0.3363	0.1996	0.2179	0.094*	
H30B	0.4014	0.2024	0.1069	0.094*	
C31	0.3221 (5)	0.3406 (4)	0.1332 (3)	0.0636 (13)	
C32	0.1919 (5)	0.3386 (5)	0.1231 (4)	0.0737 (15)	
H32	0.1485	0.2765	0.1365	0.088*	
C33	0.1260 (7)	0.4277 (6)	0.0935 (5)	0.0914 (19)	
H33	0.0374	0.4259	0.0863	0.110*	
C34	0.1867 (7)	0.5199 (6)	0.0740 (5)	0.096 (2)	
H34	0.1411	0.5806	0.0528	0.115*	
C35	0.3143 (6)	0.5212 (5)	0.0862 (4)	0.0838 (17)	
H35	0.3564	0.5838	0.0742	0.101*	
C36	0.3830 (6)	0.4332 (4)	0.1155 (4)	0.0748 (15)	
H36	0.4710	0.4359	0.1236	0.090*	
Cl1	0.75545 (12)	0.43895 (9)	0.28587 (10)	0.0660 (4)	
Cl2	0.47968 (10)	0.31446 (8)	0.39799 (8)	0.0498 (3)	
C38	0.9313 (9)	0.0002 (7)	0.7221 (8)	0.136 (3)	
N5	0.9214 (10)	−0.0854 (7)	0.7463 (8)	0.184 (4)	
C37	0.9421 (9)	0.1096 (6)	0.6906 (8)	0.165 (4)	
H37A	1.0198	0.1318	0.7091	0.198*	
H37B	0.8639	0.1434	0.7216	0.198*	
H37C	0.9495	0.1266	0.6200	0.198*	
O1W	0.6967 (15)	0.1253 (12)	0.8737 (6)	0.369 (9)	
H1WA	0.6468	0.1607	0.8369	0.554*	
H1WB	0.651 (9)	0.110 (18)	0.933 (5)	0.554*	

### Atomic displacement parameters (Å^2^)

**Table:**

	*U*^11^	*U*^22^	*U*^33^	*U*^12^	*U*^13^	*U*^23^
Cu1	0.0433 (3)	0.0369 (3)	0.0527 (4)	0.0014 (2)	−0.0148 (2)	−0.0086 (2)
O1	0.0442 (16)	0.0527 (18)	0.0462 (17)	−0.0022 (13)	−0.0043 (13)	−0.0152 (14)
O2	0.087 (3)	0.045 (2)	0.059 (2)	0.0147 (18)	−0.0079 (18)	−0.0071 (16)
O3	0.068 (2)	0.0501 (19)	0.050 (2)	0.0027 (15)	−0.0122 (15)	−0.0093 (15)
O4	0.139 (4)	0.132 (4)	0.052 (3)	0.017 (3)	−0.029 (2)	−0.029 (2)
N1	0.044 (2)	0.042 (2)	0.052 (2)	0.0022 (16)	−0.0071 (17)	−0.0099 (17)
N2	0.065 (3)	0.054 (2)	0.049 (2)	0.0007 (19)	−0.0003 (19)	−0.0117 (19)
N3	0.045 (2)	0.043 (2)	0.051 (2)	0.0040 (15)	−0.0161 (16)	−0.0106 (17)
N4	0.083 (3)	0.089 (3)	0.056 (3)	0.017 (3)	−0.030 (3)	−0.024 (2)
C1	0.056 (3)	0.067 (3)	0.066 (3)	0.009 (2)	−0.011 (2)	−0.015 (3)
C2	0.046 (3)	0.103 (5)	0.089 (4)	0.018 (3)	−0.010 (3)	−0.011 (4)
C3	0.044 (3)	0.091 (4)	0.086 (4)	−0.007 (3)	−0.024 (3)	0.004 (3)
C4	0.047 (3)	0.060 (3)	0.067 (3)	−0.009 (2)	−0.021 (2)	0.006 (2)
C5	0.071 (4)	0.092 (4)	0.072 (4)	−0.027 (3)	−0.033 (3)	−0.007 (3)
C6	0.097 (5)	0.082 (4)	0.060 (3)	−0.036 (4)	−0.028 (3)	−0.009 (3)
C7	0.082 (4)	0.065 (3)	0.051 (3)	−0.017 (3)	−0.014 (3)	−0.020 (2)
C8	0.057 (3)	0.046 (3)	0.045 (3)	−0.014 (2)	−0.013 (2)	−0.004 (2)
C9	0.046 (2)	0.044 (2)	0.048 (3)	−0.0065 (19)	−0.013 (2)	−0.002 (2)
C10	0.049 (3)	0.048 (3)	0.048 (3)	0.005 (2)	−0.003 (2)	−0.010 (2)
C11	0.053 (3)	0.042 (3)	0.047 (3)	−0.005 (2)	−0.007 (2)	−0.008 (2)
C12	0.077 (3)	0.073 (3)	0.046 (3)	−0.006 (3)	−0.006 (2)	−0.013 (3)
C13	0.084 (4)	0.056 (3)	0.045 (3)	−0.007 (3)	−0.001 (3)	−0.017 (2)
C14	0.124 (5)	0.066 (4)	0.070 (4)	−0.002 (4)	−0.030 (4)	−0.014 (3)
C15	0.153 (8)	0.076 (5)	0.107 (6)	−0.045 (5)	−0.020 (6)	0.002 (4)
C16	0.127 (7)	0.096 (6)	0.131 (7)	−0.060 (5)	0.006 (6)	−0.020 (5)
C17	0.083 (5)	0.079 (5)	0.146 (7)	−0.016 (4)	−0.013 (4)	−0.027 (5)
C18	0.080 (4)	0.055 (3)	0.093 (4)	−0.012 (3)	−0.006 (3)	−0.013 (3)
C19	0.058 (3)	0.042 (3)	0.058 (3)	0.007 (2)	−0.017 (2)	−0.013 (2)
C20	0.065 (3)	0.049 (3)	0.060 (3)	0.003 (2)	−0.019 (2)	−0.001 (2)
C21	0.063 (3)	0.039 (3)	0.079 (4)	0.003 (2)	−0.026 (3)	−0.002 (2)
C22	0.045 (3)	0.040 (3)	0.079 (4)	0.0042 (19)	−0.023 (2)	−0.011 (2)
C23	0.076 (4)	0.040 (3)	0.100 (5)	0.008 (2)	−0.026 (3)	−0.030 (3)
C24	0.088 (4)	0.054 (3)	0.091 (4)	0.010 (3)	−0.029 (3)	−0.034 (3)
C25	0.083 (4)	0.062 (3)	0.070 (4)	0.013 (3)	−0.023 (3)	−0.025 (3)
C26	0.055 (3)	0.042 (3)	0.064 (3)	0.009 (2)	−0.017 (2)	−0.017 (2)
C27	0.041 (2)	0.037 (2)	0.060 (3)	0.0052 (18)	−0.018 (2)	−0.015 (2)
C28	0.079 (4)	0.078 (4)	0.056 (3)	0.003 (3)	−0.004 (3)	−0.013 (3)
C29	0.101 (5)	0.073 (4)	0.052 (3)	0.007 (3)	−0.023 (3)	−0.013 (3)
C30	0.092 (4)	0.077 (4)	0.074 (4)	0.002 (3)	−0.033 (3)	−0.017 (3)
C31	0.074 (4)	0.075 (4)	0.047 (3)	0.005 (3)	−0.019 (2)	−0.018 (3)
C32	0.069 (4)	0.087 (4)	0.067 (4)	−0.004 (3)	−0.019 (3)	−0.011 (3)
C33	0.077 (4)	0.114 (6)	0.089 (5)	0.006 (4)	−0.024 (3)	−0.027 (4)
C34	0.107 (6)	0.100 (5)	0.084 (5)	0.028 (4)	−0.030 (4)	−0.025 (4)
C35	0.098 (5)	0.072 (4)	0.082 (4)	−0.007 (4)	−0.016 (4)	−0.016 (3)
C36	0.070 (4)	0.079 (4)	0.079 (4)	−0.005 (3)	−0.021 (3)	−0.016 (3)
Cl1	0.0627 (8)	0.0501 (7)	0.0804 (9)	−0.0065 (6)	−0.0203 (6)	0.0085 (6)
Cl2	0.0443 (6)	0.0460 (6)	0.0630 (7)	0.0010 (5)	−0.0165 (5)	−0.0138 (5)
C38	0.113 (7)	0.118 (7)	0.183 (10)	−0.006 (6)	−0.049 (6)	−0.018 (8)
N5	0.208 (10)	0.119 (7)	0.213 (10)	−0.002 (7)	−0.003 (7)	−0.038 (7)
C37	0.155 (8)	0.110 (7)	0.233 (12)	−0.035 (6)	−0.092 (8)	0.030 (7)
O1W	0.487 (18)	0.438 (17)	0.168 (8)	0.350 (16)	−0.110 (10)	−0.086 (10)

### Geometric parameters (Å, °)

**Table:**

Cu1—N1	1.993 (3)	C15—C16	1.333 (10)
Cu1—N3	2.014 (3)	C15—H15	0.9300
Cu1—Cl1	2.2364 (13)	C16—C17	1.375 (10)
Cu1—Cl2	2.2537 (13)	C16—H16	0.9300
Cu1—O1	2.423 (3)	C17—C18	1.367 (8)
O1—C8	1.360 (5)	C17—H17	0.9300
O1—C10	1.404 (5)	C18—H18	0.9300
O2—C11	1.196 (5)	C19—C20	1.384 (6)
O3—C26	1.363 (5)	C19—H19	0.9300
O3—C28	1.410 (6)	C20—C21	1.329 (7)
O4—C29	1.208 (6)	C20—H20	0.9300
N1—C1	1.299 (6)	C21—C22	1.379 (7)
N1—C9	1.357 (5)	C21—H21	0.9300
N2—C11	1.318 (5)	C22—C27	1.393 (6)
N2—C12	1.444 (6)	C22—C23	1.415 (7)
N2—H2A	0.8600	C23—C24	1.329 (7)
N3—C19	1.296 (5)	C23—H23	0.9300
N3—C27	1.367 (5)	C24—C25	1.389 (7)
N4—C29	1.311 (7)	C24—H24	0.9300
N4—C30	1.431 (7)	C25—C26	1.354 (6)
N4—H4A	0.8600	C25—H25	0.9300
C1—C2	1.399 (7)	C26—C27	1.405 (6)
C1—H1	0.9300	C28—C29	1.497 (8)
C2—C3	1.324 (8)	C28—H28A	0.9700
C2—H2	0.9300	C28—H28B	0.9700
C3—C4	1.374 (7)	C30—C31	1.497 (7)
C3—H3	0.9300	C30—H30A	0.9700
C4—C9	1.390 (6)	C30—H30B	0.9700
C4—C5	1.395 (7)	C31—C32	1.365 (6)
C5—C6	1.323 (8)	C31—C36	1.370 (6)
C5—H5	0.9300	C32—C33	1.355 (8)
C6—C7	1.392 (7)	C32—H32	0.9300
C6—H6	0.9300	C33—C34	1.362 (7)
C7—C8	1.336 (6)	C33—H33	0.9300
C7—H7	0.9300	C34—C35	1.346 (7)
C8—C9	1.410 (6)	C34—H34	0.9300
C10—C11	1.507 (6)	C35—C36	1.356 (6)
C10—H10A	0.9700	C35—H35	0.9300
C10—H10B	0.9700	C36—H36	0.9300
C12—C13	1.483 (7)	C38—N5	1.125 (10)
C12—H12A	0.9700	C38—C37	1.436 (8)
C12—H12B	0.9700	C37—H37A	0.9600
C13—C14	1.359 (7)	C37—H37B	0.9600
C13—C18	1.360 (8)	C37—H37C	0.9600
C14—C15	1.375 (9)	O1W—H1WA	0.8501
C14—H14	0.9300	O1W—H1WB	0.86 (2)
			
N1—Cu1—N3	89.95 (13)	C15—C16—C17	120.3 (7)
N1—Cu1—Cl1	89.01 (11)	C15—C16—H16	119.8
N3—Cu1—Cl1	158.68 (11)	C17—C16—H16	119.8
N1—Cu1—Cl2	162.28 (11)	C18—C17—C16	119.1 (7)
N3—Cu1—Cl2	92.14 (10)	C18—C17—H17	120.5
Cl1—Cu1—Cl2	95.29 (5)	C16—C17—H17	120.5
N1—Cu1—O1	72.19 (12)	C13—C18—C17	121.0 (6)
N3—Cu1—O1	98.37 (12)	C13—C18—H18	119.5
Cl1—Cu1—O1	101.57 (8)	C17—C18—H18	119.5
Cl2—Cu1—O1	90.10 (7)	N3—C19—C20	124.2 (4)
C8—O1—C10	117.9 (3)	N3—C19—H19	117.9
C8—O1—Cu1	110.8 (2)	C20—C19—H19	117.9
C10—O1—Cu1	131.3 (3)	C21—C20—C19	119.0 (5)
C26—O3—C28	118.1 (4)	C21—C20—H20	120.5
C1—N1—C9	118.3 (4)	C19—C20—H20	120.5
C1—N1—Cu1	118.1 (3)	C20—C21—C22	120.0 (5)
C9—N1—Cu1	123.1 (3)	C20—C21—H21	120.0
C11—N2—C12	122.7 (4)	C22—C21—H21	120.0
C11—N2—H2A	118.6	C21—C22—C27	117.9 (4)
C12—N2—H2A	118.6	C21—C22—C23	123.7 (5)
C19—N3—C27	117.1 (4)	C27—C22—C23	118.4 (5)
C19—N3—Cu1	119.6 (3)	C24—C23—C22	120.8 (5)
C27—N3—Cu1	123.3 (3)	C24—C23—H23	119.6
C29—N4—C30	123.0 (5)	C22—C23—H23	119.6
C29—N4—H4A	118.5	C23—C24—C25	121.0 (5)
C30—N4—H4A	118.5	C23—C24—H24	119.5
N1—C1—C2	122.6 (5)	C25—C24—H24	119.5
N1—C1—H1	118.7	C26—C25—C24	120.2 (5)
C2—C1—H1	118.7	C26—C25—H25	119.9
C3—C2—C1	119.0 (5)	C24—C25—H25	119.9
C3—C2—H2	120.5	C25—C26—O3	124.9 (5)
C1—C2—H2	120.5	C25—C26—C27	120.1 (4)
C2—C3—C4	120.8 (5)	O3—C26—C27	115.0 (4)
C2—C3—H3	119.6	N3—C27—C22	121.7 (4)
C4—C3—H3	119.6	N3—C27—C26	118.9 (4)
C3—C4—C9	117.6 (5)	C22—C27—C26	119.3 (4)
C3—C4—C5	123.3 (5)	O3—C28—C29	114.4 (4)
C9—C4—C5	119.2 (5)	O3—C28—H28A	108.7
C6—C5—C4	120.3 (5)	C29—C28—H28A	108.7
C6—C5—H5	119.9	O3—C28—H28B	108.7
C4—C5—H5	119.9	C29—C28—H28B	108.7
C5—C6—C7	121.4 (5)	H28A—C28—H28B	107.6
C5—C6—H6	119.3	O4—C29—N4	125.7 (6)
C7—C6—H6	119.3	O4—C29—C28	118.8 (6)
C8—C7—C6	120.2 (5)	N4—C29—C28	115.5 (5)
C8—C7—H7	119.9	N4—C30—C31	115.4 (5)
C6—C7—H7	119.9	N4—C30—H30A	108.4
C7—C8—O1	126.2 (4)	C31—C30—H30A	108.4
C7—C8—C9	120.0 (4)	N4—C30—H30B	108.4
O1—C8—C9	113.8 (4)	C31—C30—H30B	108.4
N1—C9—C4	121.7 (4)	H30A—C30—H30B	107.5
N1—C9—C8	119.3 (4)	C32—C31—C36	119.2 (5)
C4—C9—C8	119.0 (4)	C32—C31—C30	117.5 (5)
O1—C10—C11	111.9 (4)	C36—C31—C30	123.3 (5)
O1—C10—H10A	109.2	C33—C32—C31	119.7 (6)
C11—C10—H10A	109.2	C33—C32—H32	120.1
O1—C10—H10B	109.2	C31—C32—H32	120.1
C11—C10—H10B	109.2	C32—C33—C34	121.3 (6)
H10A—C10—H10B	107.9	C32—C33—H33	119.3
O2—C11—N2	124.5 (4)	C34—C33—H33	119.3
O2—C11—C10	121.8 (4)	C35—C34—C33	118.5 (6)
N2—C11—C10	113.7 (4)	C35—C34—H34	120.8
N2—C12—C13	114.3 (4)	C33—C34—H34	120.8
N2—C12—H12A	108.7	C34—C35—C36	121.5 (6)
C13—C12—H12A	108.7	C34—C35—H35	119.2
N2—C12—H12B	108.7	C36—C35—H35	119.2
C13—C12—H12B	108.7	C35—C36—C31	119.7 (5)
H12A—C12—H12B	107.6	C35—C36—H36	120.1
C14—C13—C18	118.8 (6)	C31—C36—H36	120.1
C14—C13—C12	119.6 (6)	N5—C38—C37	179.1 (12)
C18—C13—C12	121.5 (5)	C38—C37—H37A	109.5
C13—C14—C15	120.5 (7)	C38—C37—H37B	109.5
C13—C14—H14	119.8	H37A—C37—H37B	109.5
C15—C14—H14	119.8	C38—C37—H37C	109.5
C16—C15—C14	120.3 (7)	H37A—C37—H37C	109.5
C16—C15—H15	119.9	H37B—C37—H37C	109.5
C14—C15—H15	119.9	H1WA—O1W—H1WB	108.6
			
N1—Cu1—O1—C8	7.6 (3)	O1—C10—C11—O2	−19.0 (6)
N3—Cu1—O1—C8	94.8 (3)	O1—C10—C11—N2	161.3 (4)
Cl1—Cu1—O1—C8	−77.6 (3)	C11—N2—C12—C13	−88.6 (6)
Cl2—Cu1—O1—C8	−173.0 (2)	N2—C12—C13—C14	131.0 (5)
N1—Cu1—O1—C10	−175.8 (4)	N2—C12—C13—C18	−51.7 (7)
N3—Cu1—O1—C10	−88.6 (3)	C18—C13—C14—C15	−2.2 (9)
Cl1—Cu1—O1—C10	99.0 (3)	C12—C13—C14—C15	175.3 (5)
Cl2—Cu1—O1—C10	3.6 (3)	C13—C14—C15—C16	0.2 (11)
N3—Cu1—N1—C1	80.9 (4)	C14—C15—C16—C17	1.5 (13)
Cl1—Cu1—N1—C1	−77.8 (3)	C15—C16—C17—C18	−1.2 (12)
Cl2—Cu1—N1—C1	177.8 (3)	C14—C13—C18—C17	2.5 (9)
O1—Cu1—N1—C1	179.8 (4)	C12—C13—C18—C17	−174.9 (5)
N3—Cu1—N1—C9	−106.3 (3)	C16—C17—C18—C13	−0.8 (10)
Cl1—Cu1—N1—C9	95.0 (3)	C27—N3—C19—C20	−0.4 (6)
Cl2—Cu1—N1—C9	−9.4 (6)	Cu1—N3—C19—C20	176.3 (3)
O1—Cu1—N1—C9	−7.5 (3)	N3—C19—C20—C21	−0.1 (7)
N1—Cu1—N3—C19	72.2 (3)	C19—C20—C21—C22	−0.2 (7)
Cl1—Cu1—N3—C19	159.3 (3)	C20—C21—C22—C27	1.0 (7)
Cl2—Cu1—N3—C19	−90.2 (3)	C20—C21—C22—C23	−177.3 (5)
O1—Cu1—N3—C19	0.2 (3)	C21—C22—C23—C24	179.1 (5)
N1—Cu1—N3—C27	−111.3 (3)	C27—C22—C23—C24	0.7 (7)
Cl1—Cu1—N3—C27	−24.2 (5)	C22—C23—C24—C25	−0.2 (8)
Cl2—Cu1—N3—C27	86.3 (3)	C23—C24—C25—C26	1.4 (8)
O1—Cu1—N3—C27	176.7 (3)	C24—C25—C26—O3	177.4 (4)
C9—N1—C1—C2	0.0 (7)	C24—C25—C26—C27	−3.1 (7)
Cu1—N1—C1—C2	173.1 (4)	C28—O3—C26—C25	5.5 (7)
N1—C1—C2—C3	−0.8 (9)	C28—O3—C26—C27	−174.0 (4)
C1—C2—C3—C4	1.2 (9)	C19—N3—C27—C22	1.3 (6)
C2—C3—C4—C9	−0.8 (8)	Cu1—N3—C27—C22	−175.3 (3)
C2—C3—C4—C5	−179.6 (5)	C19—N3—C27—C26	−179.5 (4)
C3—C4—C5—C6	178.6 (5)	Cu1—N3—C27—C26	4.0 (5)
C9—C4—C5—C6	−0.2 (8)	C21—C22—C27—N3	−1.6 (6)
C4—C5—C6—C7	0.1 (9)	C23—C22—C27—N3	176.9 (4)
C5—C6—C7—C8	0.3 (8)	C21—C22—C27—C26	179.2 (4)
C6—C7—C8—O1	179.5 (4)	C23—C22—C27—C26	−2.4 (6)
C6—C7—C8—C9	−0.7 (7)	C25—C26—C27—N3	−175.7 (4)
C10—O1—C8—C7	−4.1 (6)	O3—C26—C27—N3	3.9 (6)
Cu1—O1—C8—C7	173.0 (4)	C25—C26—C27—C22	3.6 (7)
C10—O1—C8—C9	176.2 (3)	O3—C26—C27—C22	−176.8 (4)
Cu1—O1—C8—C9	−6.7 (4)	C26—O3—C28—C29	68.0 (6)
C1—N1—C9—C4	0.4 (6)	C30—N4—C29—O4	5.4 (9)
Cu1—N1—C9—C4	−172.3 (3)	C30—N4—C29—C28	−175.8 (5)
C1—N1—C9—C8	179.5 (4)	O3—C28—C29—O4	−150.3 (5)
Cu1—N1—C9—C8	6.7 (5)	O3—C28—C29—N4	30.9 (7)
C3—C4—C9—N1	0.0 (7)	C29—N4—C30—C31	−104.2 (6)
C5—C4—C9—N1	178.8 (4)	N4—C30—C31—C32	−171.4 (5)
C3—C4—C9—C8	−179.1 (4)	N4—C30—C31—C36	10.1 (8)
C5—C4—C9—C8	−0.2 (7)	C36—C31—C32—C33	1.6 (8)
C7—C8—C9—N1	−178.4 (4)	C30—C31—C32—C33	−177.0 (5)
O1—C8—C9—N1	1.4 (6)	C31—C32—C33—C34	−0.5 (9)
C7—C8—C9—C4	0.7 (7)	C32—C33—C34—C35	−0.9 (10)
O1—C8—C9—C4	−179.6 (4)	C33—C34—C35—C36	1.1 (10)
C8—O1—C10—C11	−67.7 (5)	C34—C35—C36—C31	0.1 (9)
Cu1—O1—C10—C11	115.9 (4)	C32—C31—C36—C35	−1.4 (8)
C12—N2—C11—O2	−2.3 (7)	C30—C31—C36—C35	177.1 (5)
C12—N2—C11—C10	177.4 (4)		

### Hydrogen-bond geometry (Å, °)

**Table:**

*D*—H···*A*	*D*—H	H···*A*	*D*···*A*	*D*—H···*A*
N2—H2A···Cl1^i^	0.86	2.35	3.160 (4)	158
N4—H4A···Cl2	0.86	2.39	3.230 (5)	165
O1W—H1WA···O2	0.85	1.95	2.776 (8)	165
O1W—H1WB···O4^ii^	0.86 (2)	2.2 (2)	2.781 (11)	121 (23)

Symmetry codes: (i) −*x*+1, −*y*+1, −*z*+1; (ii) *x*, *y*, *z*+1.

## References

[bb1] Al-Mandhary, M. R. A. & Steel, P. J. (2002). *Aust. J. Chem.* **55**, 705–708.

[bb2] Bruker (1997). *SMART* and *SAINT* Bruker AXS Inc., Madison, Wisconsin, USA.

[bb3] Sheldrick, G. M. (1996). *SADABS* University of Göttingen, Germany.

[bb4] Sheldrick, G. M. (2008). *Acta Cryst.* A**64**, 112–122. 10.1107/S0108767307043930 18156677

[bb5] Wu, W.-N., Tang, N. & Yan, L. (2008). *J. Fluoresc.* **18**, 101–107. 10.1007/s10895-007-0244-7 17917794

[bb6] Wu, G., Wang, X.-F., Okamura, T., Sun, W.-Y. & Ueyama, N. (2006). *Inorg. Chem.* **45**, 8523–8532. 10.1021/ic060493u 17029363

[bb7] Wu, W.-N., Yuan, W.-B., Tang, N., Yang, R.-D., Yan, L. & Xu, Z.-H. (2006). *Spectrochim. Acta A*, **65**, 912–918. 10.1016/j.saa.2006.01.031 16914368

[bb8] Zhu, Z., Karasawa, S. & Koga, N. (2005). *Inorg. Chem.* **44**, 6004–6011. 10.1021/ic048441x 16097820

